# Metabolomic Comparison of Patients With Colorectal Cancer at Different Anticancer Treatment Stages

**DOI:** 10.3389/fonc.2021.574318

**Published:** 2022-02-04

**Authors:** Zhuofei Li, Xingming Deng, Jun Luo, Yunpeng Lei, Xinghan Jin, Jing Zhu, Guoqing Lv

**Affiliations:** Department of Gastroinerstinal Surgery, Peking University Shenzhen Hospital, Shenzhen, China

**Keywords:** chemotherapy, colorectal cancer, metabolomics, prognostic value, surgery

## Abstract

**Background:**

The difficulties of early diagnosis of colorectal cancer (CRC) result in a high mortality rate. The ability to predict the response of a patient to surgical resection or chemotherapy may be of great value for clinicians when planning CRC treatments. Metabolomics is an emerging tool for biomarker discovery in cancer research. Previous reports have indicated that the metabolic profile of individuals can be significantly altered between CRC patients and healthy controls. However, metabolic changes in CRC patients at different treatment stages have not been explored.

**Methods:**

To this end, we performed nuclear magnetic resonance (NMR)-based metabolomic analysis to determine metabolite aberrations in CRC patients before and after surgical resection or chemotherapy. In general, a total of 106 urine samples from four clinical groups, namely, healthy volunteers (n = 31), presurgery CRC patients (n = 25), postsurgery CRC patients (n = 25), and postchemotherapy CRC patients (n = 25), were collected and subjected to further analysis.

**Results:**

In the present study, we identified five candidate metabolites, namely, N-phenylacetylglycine, succinate, 4-hydroxyphenylacetate, acetate, and arabinose, in CRC patients compared with healthy individuals, three of which were reported for the first time. Furthermore, approximately ten metabolites were uniquely identified at each stage of CRC treatment, serving as good candidates for biomarker panel selection.

**Conclusion:**

In summary, these potential metabolite candidates may provide promising early diagnostic and monitoring approaches for CRC patients at different anticancer treatment stages.

## Introduction

Colorectal cancer (CRC) is becoming a major public health concern. It is ranked as the third most frequently diagnosed cancer, making it responsible for nearly 10% of all cancer-caused deaths worldwide (1, 2). Studies have demonstrated that lifestyle (e.g., dietary risks and drinking issues), genetic (e.g., deactivation of tumor suppressor genes), and environmental (e.g., commensal microbiome) factors will greatly impact the chance of gastrointestinal tumor initiation (3–8). Unfortunately, the overall low survival rate for CRC, with a 5-year postoperative survival rate of less than 50%, is due to the high risk of tumor recurrence after surgery (9). Early localized adenomas (stage I or II) could be effectively removed by rectal resection, and over 90% of patients survived (10). For patients with late stages of CRC (stage III or IV), chemotherapy is incorporated to improve the survival rate (11). However, one group of patients did not respond to chemotherapy, and some of them developed severe side effects from this treatment (12). Thus, establishing a method to predict whether a patient will suffer the aforementioned consequences of chemotherapy would be of great value to doctors when planning timely and appropriate therapies for CRC patients.

Given that the progression of CRC is dependent on both local and systemic inflammatory responses, these indices have been used to develop CRC-specialized screening markers or scoring systems, namely, the single neutrophil-to-lymphocyte ratio (NLR), lymphocyte-to-monocyte ratio (LMR) (13–15), CRC tumor markers (CA19-9 and CEA), modified Glasgow Prognostic Score (mGPS, albumin, and C-reactive protein in serum) (16, 17), systemic inflammation score (SIS, serum albumin, and LMR) (18), and fecal-based testing (19, 20). However, the lack of sensitivity of these methods makes early diagnosis of CRC challenging (21). Therefore, the development of a more effective, noninvasive, and high-throughput method for disease-progress surveillance is necessary. In particular, metabolomics is considered a potential diagnostic method to quantitatively compare changes in low-molecular-weight compounds among different clinical groups (22). This approach has been used as a diagnostic tool for a variety of human diseases, such as cardiovascular diseases (23), diabetes (24), and respiratory disease (25), and has been extensively applied to cancer studies (26). In particular, a number of studies using tissue or biofluid samples have demonstrated changes in metabolite profiles between CRC patients and healthy controls (27). For example, significant changes in lactate, amino acids, fatty acids, carboxylic acids, and urea cycle-related metabolites have been reported when comparing tissue specimens between CRC patients and healthy controls (28–31). Furthermore, CRC morbidity was found to be associated with dysregulated glycolysis, urea cycle, tricarboxylic acid (TCA) cycle, pyrimidine and polyamine metabolism, and gut flora metabolism by using both serum and urine samples (32, 33). In addition, studies for detecting the connection between metabolomic changes and adenomatous polyps, a precursor of CRC, have been conducted (34). Intriguingly, most of these studies have been performed using serum samples (35–37) rather than urine samples (38, 39). Urine has advantages in metabolomics research due to its relatively sterile and simple context that is largely free from interfering proteins, ease in obtaining large volumes, and the existence of a large number of small molecules (over 2,650 species with a molecular weight <2,000 Da) (40).

Although an increasing number of investigations have been carried out to show the association between specific metabolites and the diagnosis of CRC (41, 42), the utilization of metabolomics for presurgical, postsurgical, and postchemotherapy comprehensive monitoring of CRC has rarely been discussed. In this study, we used a highly reproducible nuclear magnetic resonance (NMR)-based metabolomic approach to compare urine metabolic changes among four clinical groups, namely, healthy volunteers, CRC patients prior to surgery (stage II or III), and CRC patients after surgery or chemotherapy. High-resolution quantifications of over 50 metabolites were then analyzed. Their potential as diagnostic markers along with primary therapeutic intervention has been evaluated. The results from this pilot study suggested that progressive variations in urine metabolite profiles generated from an unbiased high-throughput approach could be a valuable tool for the identification of biomarkers along with the most appropriate CRC treatments.

## Materials and Methods

### Patients and Healthy Volunteers

In general, patients diagnosed with phase II/III tumor progression at the location of the ascending, descending, sigmoid, and rectal colon were enrolled for this analysis, including surgery and chemotherapy as a package. There were no comorbidities or nodal involvement in these enrolled cases. Specifically, the age of the patients ranged from 18 to 75 years, and tumor dimensions ranged from 2.8 to 6.5 cm. Furthermore, patients with other types of tumors, inflammatory bowel disease, or long-term treatment with antibiotics were excluded from this study. Subsequently, urine samples from 106 individuals from the four clinical groups were sampled in the morning, at the Peking University Shenzhen Hospital, China. For surgery and chemotherapy groups, urine samples were collected at the second day after treatment and before breakfast. Twenty-five samples were obtained from each group of CRC patients, including presurgery (Group A), postoperative (Group B), and postchemotherapy (Group C) samples. Due to several reasons, some patients could not finish both surgery and chemotherapy. Thus, additional individuals with similar clinical diagnostics and treatments were then enrolled in Groups B and C (**Table 1**). In addition, 31 samples were enrolled from healthy volunteers (Group N). For Group B patients, radical resection for CRC was performed, and for Group C patients, a chemotherapeutic protocol containing 5’-fluorouracil 400–1,250 mg/m^2^ was applied for 21 days/cycle, with 6–8 cycles per treatment according to the treatment plan of each patient. The basic characteristics of these clinical groups, such as BMI, age, sex, and diagnosis of CRC, are summarized in **Table 1**. This study was approved by the Ethics Committee of the Peking University Shenzhen Hospital, China. Informed consent was obtained from all subjects in this study.

**Table 1:** Characteristics of subjects in each clinical group.

Group	A	B	C	N
Group name	CRC-before surgery	CRC-surgery	CRC-chemotherapy	Healthy controls
BMI mean (SD)	23.0 (±3.6)	22.8 (±3.6)	22.3 (±3.2)	20.9 (±2.9)
Age mean (SD)	56.5 (±14.1)	58.5 (±12.9)	52.3 (±13.7)	52.3 (±11.4)
Men	18	18 (6)	16	21
Women	7	7 (5)	9	10
Stage II	8	11	6	/
Stage III	17	14	19	/
Total	25	25	25	31

A total of 11 patients were not treated by surgery but by chemotherapy directly. Thus, 11 new patients undergoing surgical treatment were enrolled independently in this study. The number of new patients (men or women) is indicated in brackets in Group B.

### Sample Preparation for Metabolite Extraction

Urine samples were collected and immediately frozen at −80°C to prevent disturbance until further shipment and analysis. A final concentration of 0.025% sodium azide was added to the samples prior for sample extraction. For metabolite extraction, samples were centrifuged at 13,000 rpm for 2 min, and a 450-μl aqueous layer was transferred to a clean 2-ml centrifuge tube for subsequent NMR analysis. In addition, the pH value of each aqueous layer was measured and calibrated according to the internal ion concentration database of Chenomx NMR Suit 8.1 (Chenomx Inc., Edmonton, Canada) prior to NMR analysis.

### Pretreatment of Samples and Acquisition of NMR Spectra

Generally, 50 μl of sodium trimethylsilylpropanesulfonate (DSS) standard solution (Anachro, Canada) in D_2_O was added to each sample. Samples were then mixed well before being transferred to 5-mm NMR tubes (Norell, USA). Spectra were collected using a Bruker AV III 600 MHz spectrometer (^1^H frequency: 600.13 MHz; Bruker, Germany). The first increment of a 2D-^1^H, ^1^H-NOESY pulse sequence was utilized for the acquisition of ^1^H-NMR data and for suppressing the solvent signal. Experiments used a 100-ms mixing time along with 990-ms presaturation (~80 Hz gammaB1). Spectra were collected at 25°C, with a total of 128 scans over a period of 15 min with a 12-ppm spectral width.

### Processing of NMR Spectra for Metabolite Quantification

The collected free induction decay (FID) signal was automatically zero-filled and Fourier-transformed in the processing module of Chenomx NMR Suit 8.1 (Chenomx Inc., Edmonton, Canada). The data were then carefully phased and baseline-corrected by experienced technicians in the Chenomx Processor. All spectra were referenced to the internal standard DSS and analyzed by experienced analysts against the Chenomx Compound Library. In general, a total of 75 high quality spectra were identified. Among these 75 spectra, approximately 50 metabolites were further identified and quantified (**Figure S1** and **Table S1**). All metabolite concentration information was exported to Excel and normalized by weight across all parallel samples before inclusion in the bioinformatic analysis.

### Statistics and Other Analyses Used in This Study

For quantified metabolites among the four clinical groups, a nonsupervised principal component analysis (PCA) was first carried out to observe group classification and screen potential outliers using the PCA Methods Bioconductor package. In addition, the unit-variance-scaled data were used to conduct a supervised partial least-squares discriminant analysis (PLS-DA) using the PLS package for the maximization of group variations. Furthermore, parameters such as interpretability (R2) and predictability (Q2) were used to monitor the quality of the proposed model. Primary metabolites were selected to view metabolite shifts among groups. Subsequently, a third random forest test was conducted for further comparisons using the randomForest R package. Additionally, after testing the datasets for normality, two-tailed Student’s t test was used as well for group comparisons between groups using SPSS 19.0 (IBM; USA). Significant findings between the two groups were determined using a *P*-value <0.05 after adjustment for multiple comparisons. Plots were made using the ggplot2 package, and the Ward method was used for clustering analysis, as shown in **Figure 2** and **Figure S2**. Finally, pathway enrichment analysis was carried out using MetaboAnalyst tools to locate the overrepresented pathways between the two comparison groups (43).

## Results

### Characteristics of Clinical Groups and Experimental Setup

In this study, four clinical groups were designed for metabolomic analysis. Three groups of CRC patients were compared with 31 healthy individuals (Group N). Specifically, Group A contained 25 individuals diagnosed with stage II or III CRC before surgical resection. The postsurgery group (Group B) enrolled 25 CRC patients who were treated through surgical procedures. Group C was a group of 25 CRC patients who underwent chemotherapy treatment after surgery (**Table 1**). The initial aim of this study was to observe the metabolic changes in CRC patients along with CRC treatments. However, for several reasons, some individuals could not finish the entire treatment for sample collection. Alternatively, an additional 11 patients with similar CRC conditions and surgical treatment procedures were enrolled in Group B to calculate the corresponding parameters (age, sex, and CRC stages, among others), are shown in **Table 1**.

**Figure 1:**
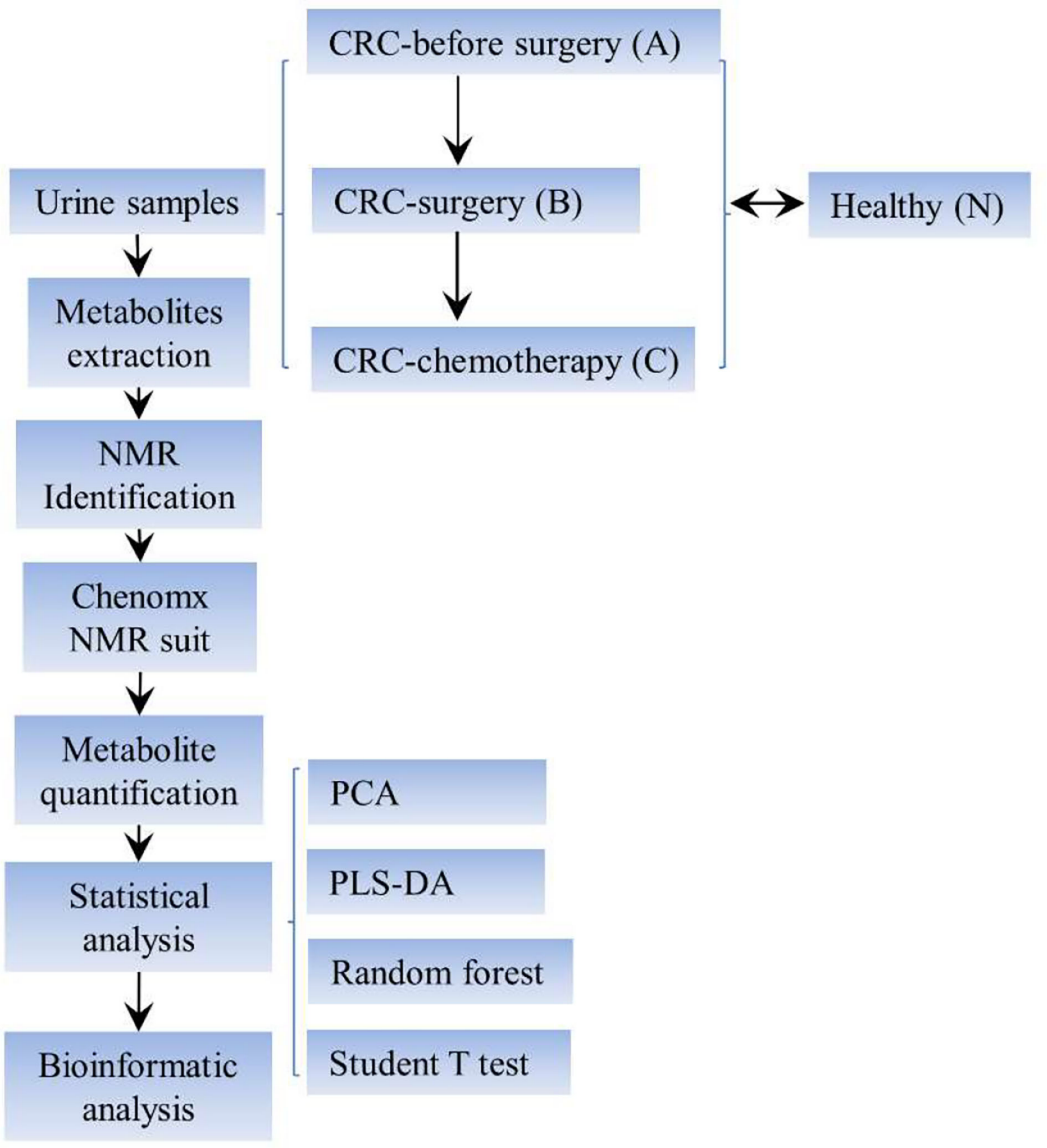
Schematic view of the experimental setup of this study. CRC, colorectal cancer; NMR, nuclear magnetic resonance; PLS-DA, partial least squares discriminant analysis.

The overall experimental design is summarized in **Figure 1**. Urine samples were collected from the aforementioned clinical groups for metabolite extraction and subsequent NMR identification. The spectral data were further analyzed using the Chenomx NMR suite for metabolite quantification purposes. Furthermore, a series of statistical analyses, such as a nonsupervised principal component analysis (PCA), a multivariate statistical method denoted as supervised partial least-squares discriminant analysis (PLS-DA), random forest test, and Student’s t test, were conducted to unravel the potential variations and biomarker discovery among the four clinical groups. 

### Characteristics of Metabolite Profiling and Quantification

**Figure 2:**
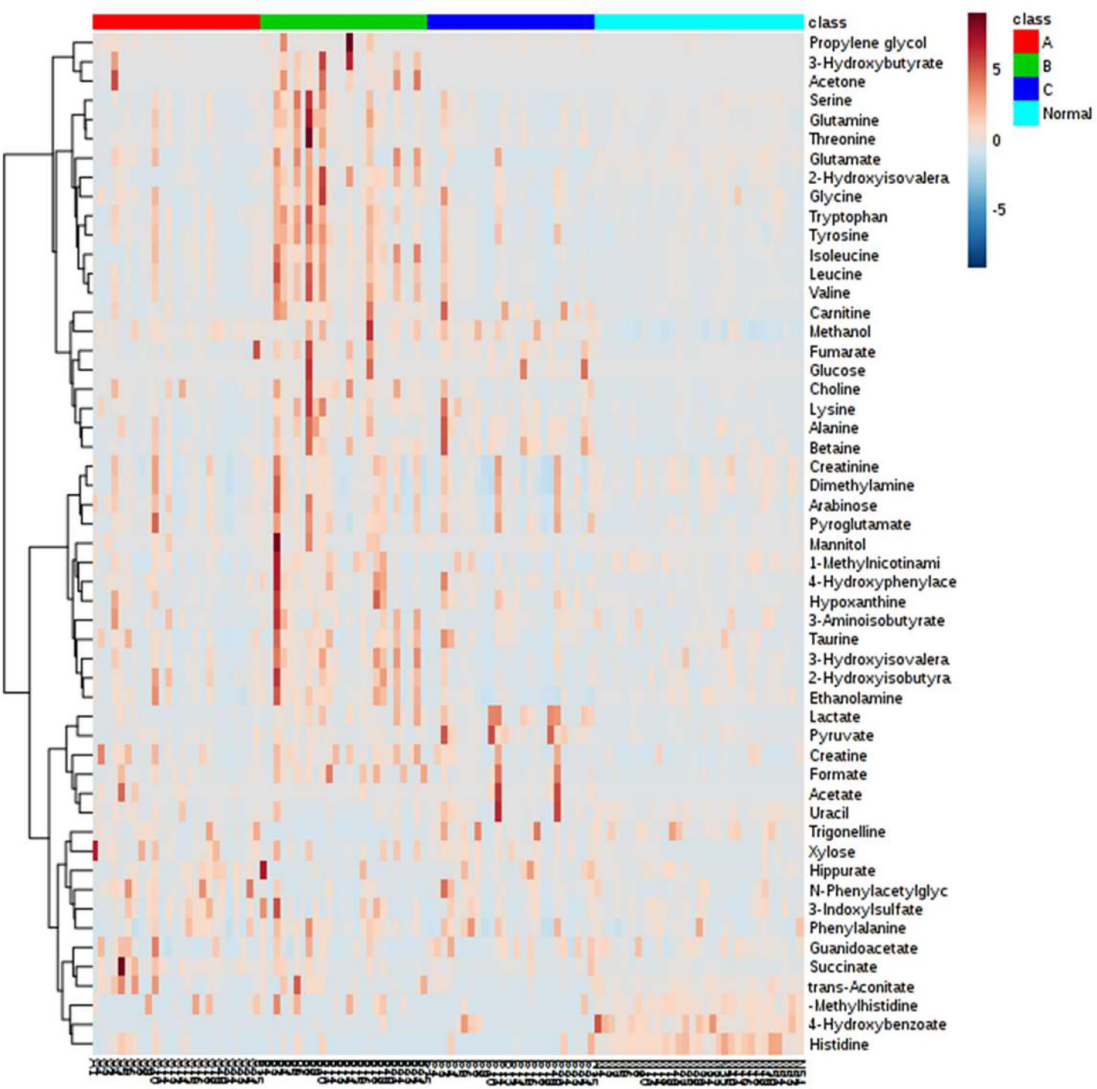
Heatmap representation of all metabolites detected in this study among the four clinical groups. Metabolites with similar profiles among the four clinical groups are clustered on the y-axis of this heatmap.

In general, over 50 metabolites belonging to diverse metabolic classes, namely, amino acids and derivatives, amine and ammonium compounds, organic acids, sugars, alcohols, nucleic acid components, and other cofactors, were identified in the urine samples from these 106 individuals (**Table S1**). The overall amount of each metabolite is presented in the form of a heatmap for each individual (**Figure 2**). Clustering analysis suggested that healthy individuals have a similar composition of urine metabolites, whereas different groups of CRC patients have large variations in metabolite distribution or abundance (**Figure S2**).

### Statistical Analysis of Metabolic Profiles Among Clinical Groups

**Figure 3:**
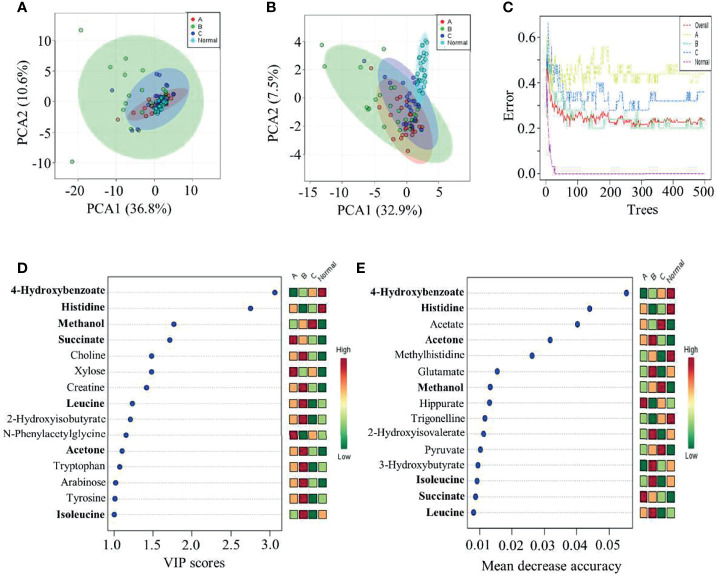
Statistical comparison among the four clinical groups. **(A)** PCA, **(B, D)** PLS-DA, and **(C, E)** random forest tests were performed to identify group characteristics and potential metabolite biomarkers along with CRC treatment. Group A, CRC patients before surgery; Group B, CRC patients after surgery; Group C, CRC patients after chemotherapy; and Group N, healthy control group.

To further reveal the features of metabolic profiles among the four clinical groups, multiple statistical analyses were performed on this dataset (**Figure 3**). The initial PCA did not suggest a difference between healthy individuals and the three groups of CRC patients (**Figure 3A**). Thus, a supervised partial least-squares PLS-DA method for multivariate analysis was subsequently conducted. The results of this analysis indicated that the metabolic profiles of healthy volunteers were distinct from those of CRC patients in the remaining three groups (**Figure 3B**). Meanwhile, postoperative patients were more diverse in their metabolic profiles than the CRC patients in the other two groups (**Figure 3B**). In addition, a third method, that is, random forest estimation, was applied to show similar separation patterns between the three CRC groups and healthy individuals. Subsequently, the top 15 metabolites showing significant differences among the four clinical groups were presented using PLS-DA and random forest approaches (**Figures 3D, E**). Intriguingly, seven of these metabolites could be found on both lists, suggesting that statistical analysis may cause considerable variation in determining the target of interest. Because PLS-DA is a universal method used for metabolic profiling, subsequent analyses were based on this method. Additional Student’s t-tests were also applied for subsequent group comparisons.

### Group Comparisons Between Clinical Groups Identified Specific Metabolites as Potential Biomarkers for CRC Patients

**Figure 4:**
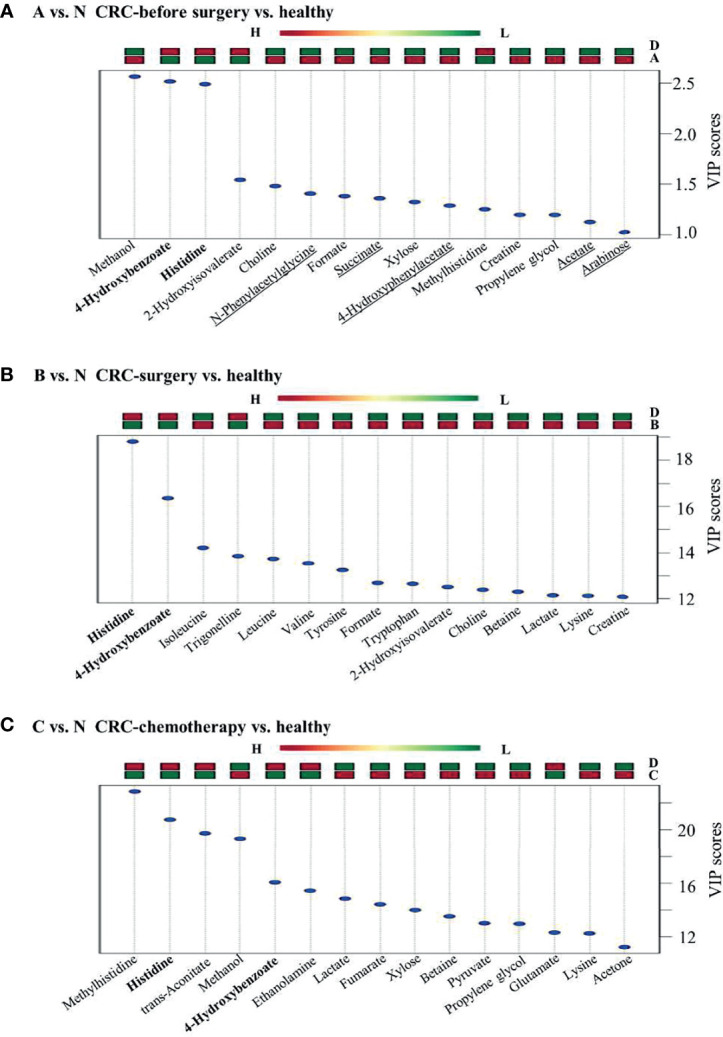
Group comparisons among the three pairs of clinical groups. **(A)** CRC patients versus healthy controls, **(B)** CRC surgery versus healthy controls, and **(C)** CRC chemotherapy versus healthy controls. Metabolites found in all three comparison groups are shown in bold. Metabolites uniquely identified in panel **(A)** are underlined. Up or downregulation of each metabolite is presented above the figure by using color bars.

Group comparisons for differentially accumulated metabolites between various CRC groups and healthy individuals were made to determine potential biomarkers associated with CRC. In particular, two metabolites, 4-hydroxybenzonate and histidine, were consistently present in all three comparison groups (e.g., A vs. N, B vs. N, and C vs. N) (**Figure 4**), showing low abundance in the groups of CRC patients at various treatment stages. In contrast, several metabolites, namely, N-phenylacetylglycine, succinate, 4-hydroxyphenylacetate, acetate, and arabinose, were uniquely upregulated in presurgery CRC patients versus healthy individuals, indicating their potential value in CRC biomarker discovery. However, validations of these biomarkers are needed using a larger cohort. Furthermore, the VIP scores of B vs. N and C vs. N were much larger than those of A vs. N, suggesting significant changes in certain metabolites in these two comparison groups. In addition, it is known that after surgery cancer biomarkers can change as a consequence, which is consistent in our findings. However, what are the potential usage of these identified metabolites remains to be further investigated.

### Comparisons Between Untreated and Treated CRC Groups Identified Specific Metabolites Associated With CRC Therapy

**Figure 5:**
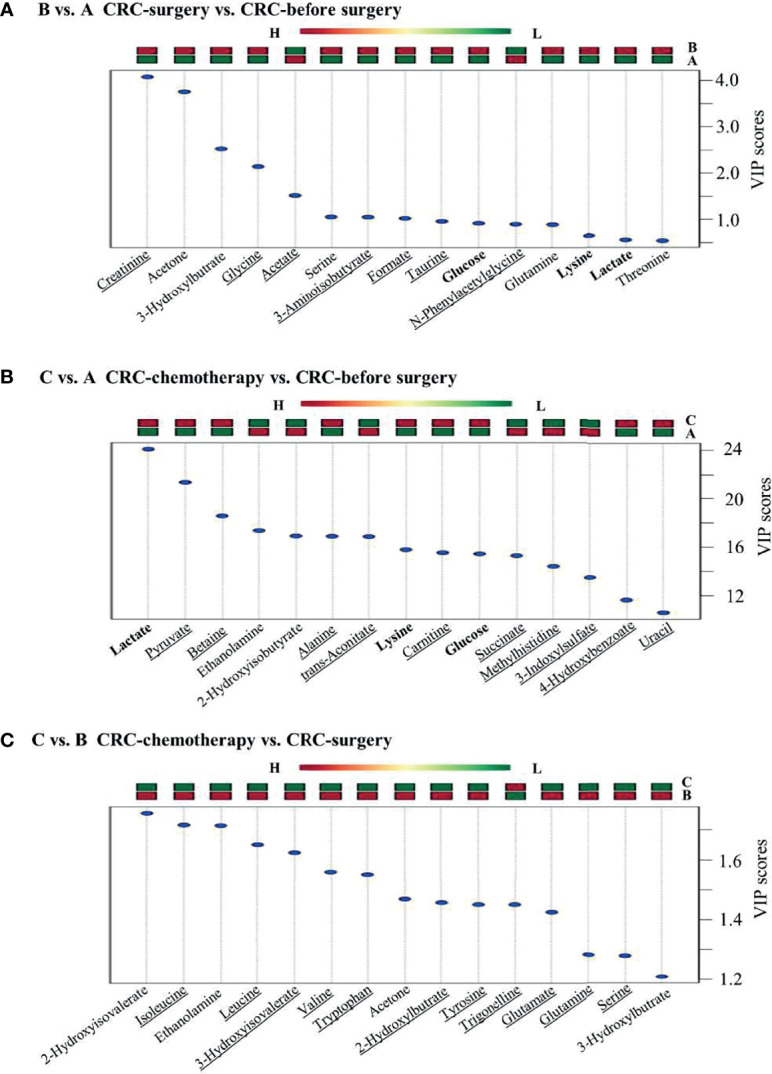
Group comparisons between the two groups alone with CRC clinical treatment. **(A)** CRC patients before surgery versus healthy controls, **(B)** CRC patients after surgery versus before surgery, and **(C)** CRC patients after chemotherapy versus after surgery.

To further understand the metabolic changes during CRC treatments, group comparisons between CRC patient groups at various treatment stages were conducted. Generally, each comparison group showed a distinct pattern of metabolic change. Many metabolites were uniquely present in each comparison group (**Figure 5**). For example, 9 out of 15 metabolites from B vs. A, 10 out of 15 metabolites from C vs. A, and 11 out of 15 metabolites from C vs. B were uniquely regulated in each comparison group (**Figure 5**), suggesting that specific metabolic features are associated with different treatment stages of CRC. Specifically, three metabolites, namely, glucose, lysine, and lactate, were upregulated after either surgical resection or chemotherapy compared with CRC patients before treatment (**Figures 5A, B**), implying the possible value of these metabolites to indicate the effectiveness of CRC treatments. Specifically, the presence of D-lactate and glucose is commonly linked to diabetes. The underlying mechanism of the elevation of these metabolites in both treatment groups remains to be elucidated. Furthermore, histidine was characterized in three previous comparison groups against healthy controls, whereas this metabolite was not discriminant in comparison groups such as B vs. A and C vs. A, suggesting its potential as a CRC biomarker to differentiate CRC patients from healthy individuals. The authenticity of these metabolites to serve as biomarkers will be evaluated in large population studies from multiple centres in the future.

### Unique Metabolic Pathways Were Enriched Between Different Comparison Groups

**Figure 6:**
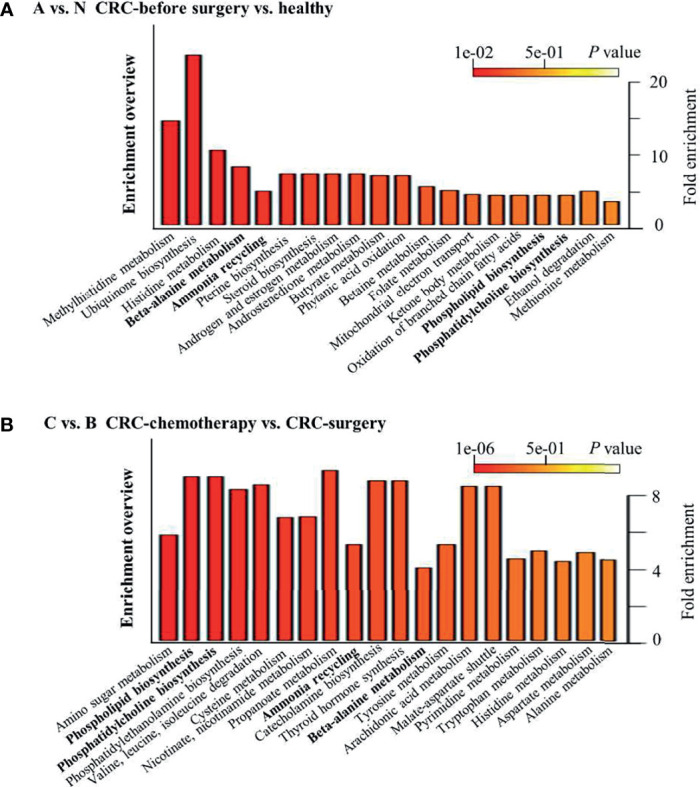
Top 20 enriched pathways between selected clinical groups. **(A)** CRC patients versus healthy controls and **(B)** CRC patients after chemotherapy versus patients after surgery.

Pathway enrichment analysis was subsequently performed to elucidate representative metabolic pathways among different comparison groups. Similar to the metabolite changes, different metabolic pathways were enriched in comparison groups, such as A vs. N and C vs. B (**Figure 6**), suggesting that the differentially regulated metabolites detected in each comparison group belong to different metabolic pathways. In particular, among the top 20 enriched pathways, only four were present in both aforementioned comparison groups: beta-alanine metabolism, ammonia recycling, phospholipid biosynthesis, and phosphatidylcholine biosynthesis. In addition, no enrichment results were obtained from comparison groups B vs. A (**Figure 5A**).

## Discussion

Along with the development of metabolomic methods, novel perspectives, such as early CRC diagnosis and monitoring of treatment procedures, can be offered by clinicians (44). The current investigation examined urine metabolic profiles as an approach for evaluating potential biomarker combinations for the early diagnosis of CRC and patient responses to radical resection or chemotherapy. The goal of this study was to obtain preliminary insights into metabolic variations during the aforementioned situations, providing an additional tool for clinical counseling. 

### Metabolomic Data Obtained in Different Treatment Stages Facilitate Biomarker Ddiscovery in CRC Patients

In this study, candidate metabolites for CRC patients at different stages of treatment (presurgery and postsurgery) were chosen according to PLS-DA analysis. Interestingly, we found that the incorporation of other stages of CRC treatment could eliminate false-positive identification in biomarker discovery. For example, a previous study demonstrated that higher activity of histidine decarboxylase might decarboxylate histidine, thus decreasing its level in CRC patients in comparison to healthy control groups (22, 45). However, the level of histidine was observed to be significantly altered in both comparison groups, that is, the postsurgery and postchemotherapy groups, suggesting that it is not a good candidate for biomarker discovery in CRC. Furthermore, opposite results of histidine alteration between the postsurgery and presurgery groups were documented in urine samples of CRC patients (33), suggesting that clinical procedures such as nutritional supplementation of amino acids may affect urine metabolic profiles. Thus, the selection of metabolites that are uniquely present in presurgery CRC patients versus healthy controls is a better approach towards biomarker discovery in patients. Here, five metabolites, namely, N-phenylacetylglycine, succinate, 4-hydroxyphenylacetate, acetate, and arabinose, were upregulated in CRC patients compared with healthy individuals, and none of them were present in the other comparison groups. In particular, besides succinate and hydroxyphenylacetate, N-phenylacetylglycine, acetate, and arabinose have not been reported previously, indicating their potential role as biomarkers of CRC. Furthermore, succinate from the tricarboxylic acid cycle has long been identified as being associated with CRC (38), suggesting the validity of our approach for biomarker screening.

In addition to biomarker discovery between CRC patients and healthy volunteers, the metabolic profiles of CRC patients at different treatment stages provided valuable information for monitoring the efficacy of a certain treatment, such as radical resection or chemotherapy. First, three metabolites (glucose, lysine, and lactate) identified in comparison groups C vs. N and B vs. N (**Figures 5A, B**) served as common metabolic candidates for cancer treatment. Higher glucose levels have been reported previously in serum samples of CRC patients (46). In our study, the level glucose was increased in CRC patients after either surgical resection or chemotherapy. Furthermore, consistent with a previous report (47), lysine was consistently found to be at a lower level in presurgery CRC patients than in patients after surgery. Our study also suggested that chemotherapy could affect lysine levels in urine samples through a similar pattern of surgical operation. In addition, elevated lactate levels have been observed in numerous tumour tissues (32). However, both surgical resection and chemotherapy were able to further increase lactate levels, which requires further investigation. Nevertheless, the reliability of these metabolic indicators also requires further examination in a large-scale study. Additionally, nine and ten metabolites were identified as unique features of B vs. A and C vs. A, respectively (**Figures 5A, B**). Some of these metabolites, namely, creatinine, 3-aminoisobutyrate, and taurine, were first reported to be associated with CRC and its different treatment stages, providing novel targets for further investigation.

### Factors Affecting Urine Metabolic Profiles in CRC Patients

Tumor tissues have been demonstrated to have higher metabolic status, thus affecting numerous primary metabolic processes. Some of these variations can be detected in urine samples. The significantly changed metabolites identified in this study are more likely the result of altered metabolic status in CRC patients compared with healthy individuals. However, other factors may also influence the urine metabolic profile in CRC patients. For example, tyrosine identified in comparison group C vs. B (**Figure 5C**) may serve as a precursor for p-cresol and hydroxyphenylacetate, which are aromatic compounds involved in microbial metabolism (e.g., in *Clostridium difficile*) (47, 48). *Clostridium* spp. are common gut commensal microbes in healthy individuals (10). Intriguingly, 4-hydroxyphenylacetate was identified as a potential biomarker for CRC in this study, suggesting the involvement of gut microbiota in affecting urine metabolite composition. Furthermore, both the procedures involved in surgical operation and the chemicals used in chemotherapy have a large impact on the gut ecosystem microbial composition. Thus, the relationship between urine metabolomics and gut microbiome variation associated with CRC patients would be an interesting research topic in the future.

### Limitations of the Statistics and Analytical Methods Used in This Study

One important thing we need to discuss is that the application of a statistical model will greatly affect the final outcomes for feature metabolite determination. For example, PLS-DA and random forest analysis provided two lists of significantly changed metabolites among the four clinical groups (**Figures 3D, E**). Furthermore, only 7 out of 15 compounds were present on both lists. Specifically, PLS-DA is a supervised method that can improve group separation for biomarker identification (49). It has been used in numerous metabolomic studies for biomarker screening in CRC patients (33, 38). Although both methods could separate healthy individuals from CRC patients at different treatment stages, discrepancies were observed in the final list of candidate metabolites. Furthermore, we also used two methods, namely, PLS-DA and Student’s t-test, to analyze the group comparison data (**Table S2**). Interestingly, the outcomes of significantly altered metabolites were almost the same in some comparison groups, whereas some comparison groups showed a completely different metabolite list obtained by these two methods. Thus, the underlying mechanism of these phenomena remains to be elucidated. Studies aiming to address this issue would be greatly helpful for future metabolic biomarker identification. Furthermore, in addition to the small sample size and relatively low sensitivity of NMR spectrometry (33, 50), the present study could be further improved by incorporating other omics approaches, namely, proteogenomics (51, 52), metagenomics (53, 54), and proteomics (55), to further expand the functional relationship between these metabolites and host metabolic functions.

## Conclusion

In this pilot study, we identified a panel of potential biomarkers from urine metabolites of Chinese CRC patients obtained at different treatment stages. Some of these compounds were reported for the first time in CRC studies. These outcomes reveal the value of this noninvasive and high-throughput strategy as a complementary diagnostic and monitoring tool for CRC. We hope the metabolites identified here could be developed into a simple urine test that is applicable in clinical practice, thus having a major salutary effect on CRC mortality.

## Data Availability Statement

The original contributions presented in the study are included in the article/**Supplementary Material**. Further inquiries can be directed to the corresponding author.

## Ethics Statement

The studies involving human participants were reviewed and approved by the Peking University Shenzhen Hospital. The patients/participants provided their written informed consent to participate in this study.

## Author Contributions

GL designed the experiments, and ZL and XD conducted them. JL, JZ, YL and XJ analyzed the data. ZL wrote the manuscript, and GL critically commented and revised it.

## Funding

This work was supported by grants from the San Ming Project of Shenzhen, China, and the Municipal Health Planning Commission Fund of Shenzhen, China (No. SZXJ 2018084). 

## Conflict of Interest

The authors declare that the research was conducted in the absence of any commercial or financial relationships that could be construed as a potential conflict of interest.

## Publisher’s Note

All claims expressed in this article are solely those of the authors and do not necessarily represent those of their affiliated organizations, or those of the publisher, the editors and the reviewers. Any product that may be evaluated in this article, or claim that may be made by its manufacturer, is not guaranteed or endorsed by the publisher.
