# Supplementary material for: Metabolomic Comparison of Patients With Colorectal Cancer at Different Anticancer Treatment Stages
Source: Front Oncol. 2022 Feb 4;11:574318. doi: 10.3389/fonc.2021.574318 (PMC8855116; doi:10.3389/fonc.2021.574318)
Supplement: Supplementary file 1 [file Image_1.pdf]

Title: Metabolomic comparison of patients with colorectal cancer at different anti-cancer treatment stages

Running title: Metabolic profiling of CRC patients

Zhuofei Li[1], Xingming Deng[1], Jun Luo[1], Yunpeng Lei[1], Xinghan Jin[1], Guoqing Lv[1]

[1] Department of Gastroinerstinal Surgery, Peking University Shenzhen Hospital, ShenZhen 518033, PR China.

Corresponding authors

Guoqing Lv

Department of Gastroinerstinal Surgery, Peking University Shenzhen Hospital, ShenZhen 518033, PR China.

lgq_bdsy@163.com

Figure S1 Representative spectra of urine samples

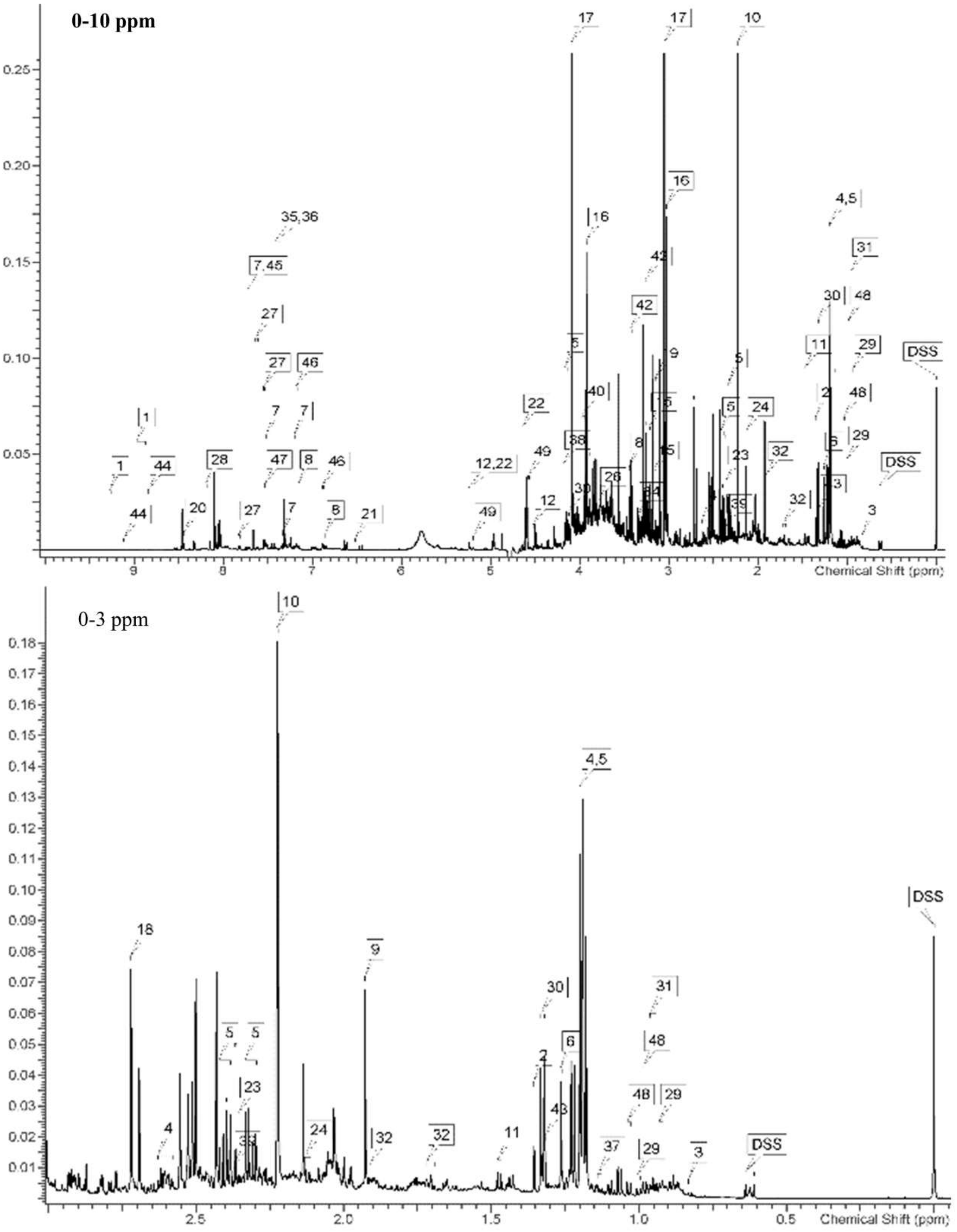

Figure S1 Representative spectra of urine samples continued

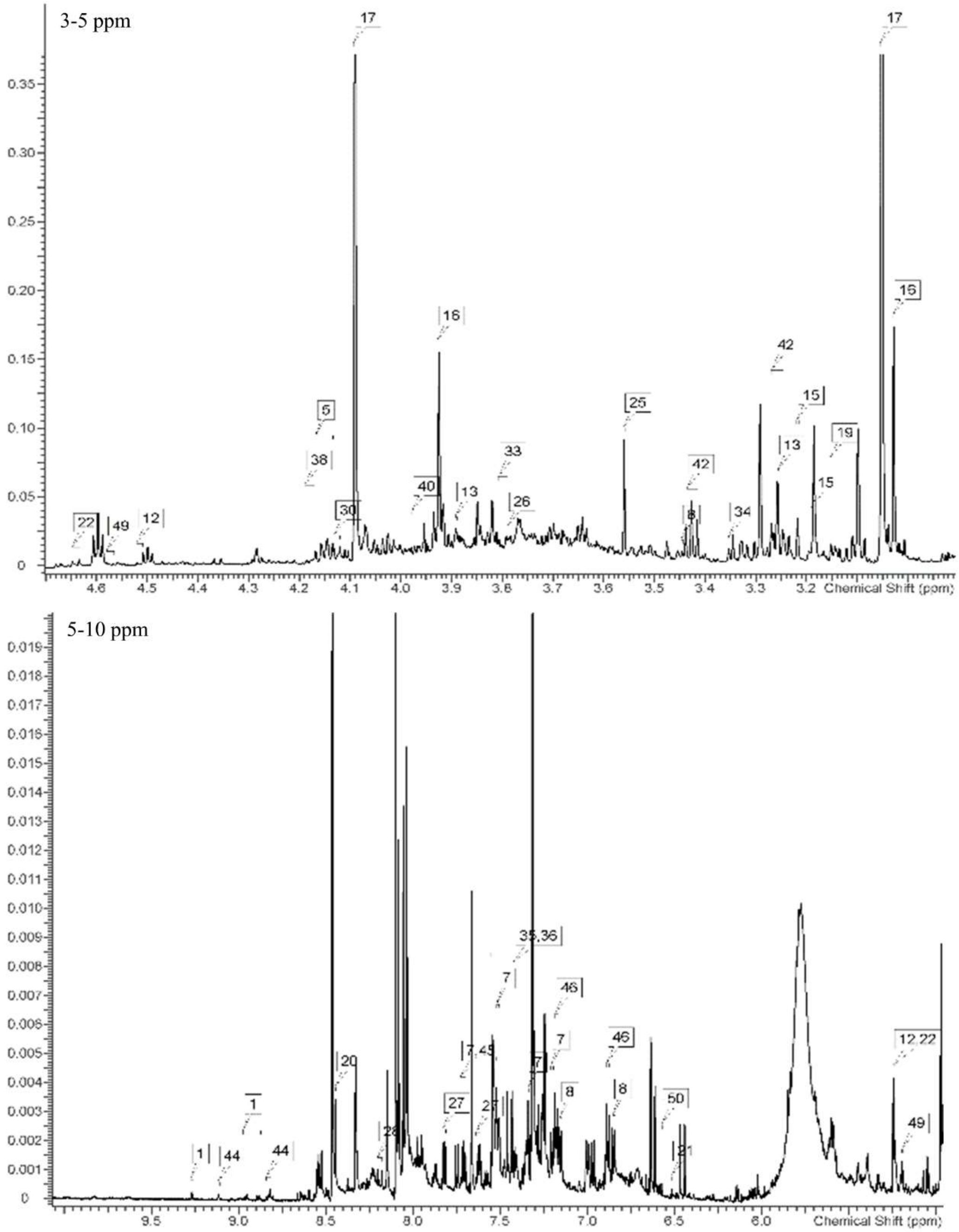

Figure S1 Representative spectra of selected metabolites. NMR spectra are presented at different detection ranges including total range 0-10 ppm, subranges 0-3, 3-5 and 5-10 ppm. The corresponding metabolite of each number is summarized in Table S1.

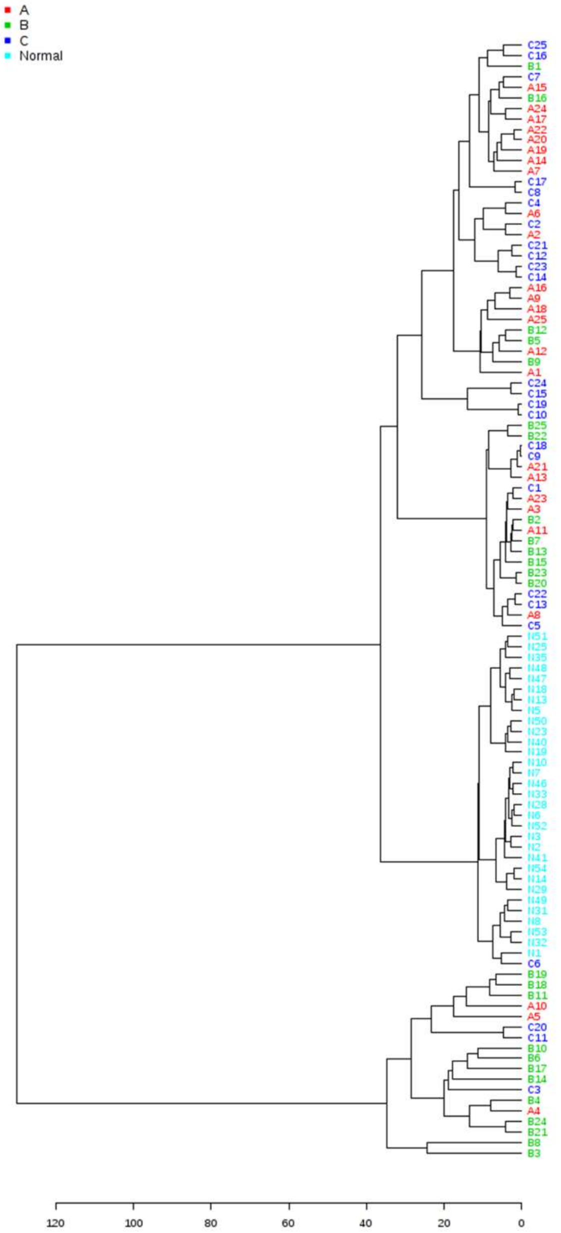

Figure S2 Cluster analysis of all samples in this study. Group A, pre-surgery CR patients; Group B, post-operative CRC patients; Group C, post-chemotherapy CRC patients; Group N, healthy individuals.
