# Supplementary material for: Metabolomic Comparison of Patients With Colorectal Cancer at Different Anticancer Treatment Stages
Source: Front Oncol. 2022 Feb 4;11:574318. doi: 10.3389/fonc.2021.574318 (PMC8855116; doi:10.3389/fonc.2021.574318)
Supplement: Supplementary file 2 [file Table_1.pdf]

Table S1 Information of metabolites detected by NMR

Metabolites	Spectrum No.	Class
1-Methylnicotinamide	1	vitamin/cofactors
2-Hydroxyisobutyrate	2	organic acids
2-Hydroxyisovalerate	3	organic acids
3-Aminoisobutyrate	4	amino acids
3-Hydroxybutyrate	5	organic acids
3-Hydroxyisovalerate	6	organic acids
3-Indoxylsulfate	7	amino acid derivatives
4-Hydroxyphenylacetate	8	organic acids
Acetate	9	organic acids
Acetone	10	ketones
Alanine	11	amino acids
Arabinose	12	sugars
Betaine	13	amino acids
Carnitine	14	amino acids
Choline	15	ammoniums compounds
Creatine	16	amino acid derivatives
Creatinine	17	amines
Dimethylamine	18	amines
Ethanolamine	19	amines
Formate	20	organic acids
Fumarate	21	organic acids
Glucose	22	sugars
Glutamate	23	amino acids
Glutamine	24	amino acids
Glycine	25	amino acids
Guanidoacetate	26	amino acid derivatives
Hippurate	27	amino acid derivatives
Hypoxanthine	28	nucleic acid components
Isoleucine	29	amino acids
Lactate	30	organic acids
Leucine	31	amino acids
Lysine	32	amino acids
Mannitol	33	sugars
Methanol	34	alcohols
N-Phenylacetylglycine	35	amides
Phenylalanine	36	amino acids
Propylene glycol	37	alcohols
Pyroglutamate	38	amino acid derivatives
Pyruvate	39	organic acids
Serine	40	amino acids
Succinate	41	organic acids
Taurine	42	amino acids
Threonine	43	amino acids
Trigonelline	44	food and drug compounds
Tryptophan	45	amino acids
Tyrosine	46	amino acids
Uracil	47	nucleic acid components
Valine	48	amino acids
Xylose	49	sugars
trans-Aconitate	50	organic acids
